# Predicting the Outcome of Patients with Severe COVID-19 with Simple Inflammatory Biomarkers: The Utility of Novel Combined Scores—Results from a European Tertiary/Referral Centre

**DOI:** 10.3390/jcm13040967

**Published:** 2024-02-08

**Authors:** Maria P. Ntalouka, Alexandros Brotis, Maria Mermiri, Athanasios Pagonis, Athanasios Chatzis, Metaxia Bareka, Paraskevi Kotsi, Ioannis Pantazopoulos, Konstantinos Gourgoulianis, Eleni M. Arnaoutoglou

**Affiliations:** 1 Department of Anesthesiology, Faculty of Medicine, School of Health Sciences, University of Thessaly, Larissa University Hospital, 41110 Larissa, Greece; maria.ntalouka@icloud.com (M.P.N.); mmermiri@gmail.com (M.M.); thanasischatzis94@gmail.com (A.C.); barekametaxia@hotmail.com (M.B.); 2 Department of Neurosurgery, Faculty of Medicine, School of Health Sciences, University of Thessaly, Larissa University Hospital, 41110 Larissa, Greece; alexgbrodis@yahoo.com; 3 Department of Respiratory Medicine, Faculty of Medicine, University of Thessaly, 41110 Larissa, Greece; thanos.pgns@gmail.com (A.P.); pantazopoulosioannis@yahoo.com (I.P.); kgourg@uth.gr (K.G.); 4 Department of Transfusion Medicine, University of Thessaly, 41110 Larissa, Greece; vkotsis@uth.gr; 5 Department of Emergency Medicine, Faculty of Medicine, University of Thessaly, 41110 Larissa, Greece

**Keywords:** coronavirus disease 2019, COVID-19, lymphocyte subsets, C-reactive protein, biomarkers, prognosis, severe acute respiratory syndrome

## Abstract

**Background:** The clinical significance of combinations of inflammatory biomarkers in severe COVID-19 infection is yet to be proved. Although several studies have evaluated the prognostic value of biomarkers in patients with COVID-19, there are limited data regarding the value of the combination scores that could take full advantage of the prognostic value of several biomarkers and that could account for the heterogeneity of patients with severe COVID-19. We investigated the prognostic value of combination scores of admission values of inflammatory biomarkers in adults with severe COVID-19. **Methods:** Adults admitted to the Department of Respiratory Medicine of the UHL with severe COVID-19 (April-September 2021, NCT05145751) were included. Demographics, medical history, laboratory tests and outcome (high-flow nasal cannula (HFNC), admission to Intensive Care Unit (ICU) or death) were recorded. The optimal cut-off points of on admission values of C-reactive protein (CRP), CRP to lymphocyte ratio (CLR), lymphocyte to neutrophil ratio (LNR) and derived variation of neutrophil to lymphocyte ratio (dv-NLR (neutrophil/white blood count-lymphocyte)) for the predetermined outcome were defined. Based on the cut-off of CRP, LNR, dv-NLR and CLR, which were found to be predictors for HFNC, 3 scores were defined: CRP and LNR (C-CRP #1), CRP and dv-NLR (C-CRP #2), CRP and CLR (C-CRP #3). Likewise, based on the cut-off of CRP and CLR, which were found to be predictors for death, the score of CRP and CLR (C-CRP #3*) was defined. The combination scores were then classified as: 2 points (both biomarkers elevated); 1 point (one biomarker elevated) and 0 points (normal values). None of the biomarkers was predictive for the ICU admission, so no further analysis was performed. Binomial logistic regression analysis was used to establish the predictive role for each biomarker. **Results:** One hundred and fifteen patients (60% males, mean age 57.7 years) were included. Thirty-seven (32.2%) patients required HFNC, nine (7.8%) died and eight (7%) were admitted to ICU, respectively. As far as HFNC is concerned, the cut-off point was 3.2 for CRP, 0.231 for LNR, 0.90 for dv-NLR and 0.004 for CLR. Two points of C-CRP #1 and 2 points of C-CRP #3 predicted HFNC with a probability as high as 0.625 (*p* = 0.005) and 0.561 (*p* < 0.001), respectively. Moreover, 1 point of C-CRP #2 and 2 points of C-CRP #2 predicted HFNC with a probability of 0.333 and 0.562, respectively. For death, the optimal cut-off point for CRP was 1.11 and for CLR 3.2*1033. Two points of C-CRP #3* with an accuracy of 0.922 predicted mortality (*p* = 0.0038) in severe COVID-19. **Conclusions:** The combination scores of CRP and inflammatory biomarkers, based on admission values, are promising predictors for respiratory support using HFNC and for mortality in patients suffering from severe COVID-19 infection.

## 1. Introduction

Coronavirus disease 2019 (COVID-19) has led to increased morbidity and mortality and was declared a public health emergency of international concern by the World Health Organization (WHO) in January 2020 [1]. COVID-19 has been characterized as a highly contagious disease that mostly affects the respiratory system and has been held responsible for one of the longest pandemics in world history [2].

The majority of patients, up to 80%, may experience mild symptoms such as nasal congestion, sore throat, headache, fatigue, muscle pain, fever or persistent cough [3]. However, a subset of patients may experience a massive inflammatory response, known as a “cytokine storm”, which has been recognized as a significant poor prognostic factor [4,5,6,7]. This dysregulated, maladaptive and excessive cytokine release is quite complex and has been linked with disease progression and increased morbidity and mortality. It is characterized by loss of regulatory control of proinflammatory cytokine production, locally and systemically, and it leads to acute respiratory distress syndrome (ARDS) and/or multiple-organ failure, with increased risk for escalation of respiratory support or intubation, mechanical ventilation and intensive care unit (ICU) admission or death [3,4,5,6,7].

Given the rapid spread of the virus and the increased risk of life-threatening complications, an optimum and timely diagnosis and treatment of severe disease manifestations are of utmost importance. To date, several simple, inexpensive and easy and fast to be obtained inflammatory biomarkers have been used for predicting the severity and progression of COVID-19 disease, such as neutrophil to lymphocyte ratio (NLR), derived neutrophil to lymphocyte ratio (dNLR), monocyte to lymphocyte ratio (MLR) and platelet to lymphocyte ratio (PLR). In addition, CRP has been associated with disease severity since the early stages of the pandemic. Overall, obtaining the levels of the aforementioned biomarkers upon admission could allow stratification of risk in COVID-19 patients and early identification of high-risk patients with severe disease. However, the clinical relevance and utility of a new blood score that combines different simple biomarkers, such as CRP and LNR, and takes full advantage of their prognostic value has not yet been assessed in patients with severe COVID-19 disease, and further research is needed to identify the best-fit predictive biomarker [4,5,6,7,8]. Moreover, it has been suggested that the heterogeneity of patients with COVID-19 accentuates the need for implementation of multiple biomarkers in order to ideally evaluate the dynamic course of the disease and guide the stratification of patients with severe COVID-19 infection.

The purpose of the present study was to investigate the prognostic value of admission values and combination scores of simple inflammatory biomarkers in predicting the outcome, in terms of (i) escalation of respiratory support with the use of high-flow nasal cannula (HFNC), (ii) admission to Intensive Care Unit (ICU) or (iii) death, in patients with severe COVID-19 infection.

## 2. Materials and Methods

### 2.1. Study Design

This study is a retrospective analysis of prospectively collected electronic data. Data were collected from the electronic system of our hospital during the period between April 2021 and September 2021, which coincided with the period of the Delta variant wave of the COVID-19 pandemic. This is a sub-analysis of a study protocol that was approved by the Ethical Committee of our institution (42937, 29 November 2021) and submitted on Clinicaltrials.gov (registration number: NCT05145751) [8]. Since the collection of patient data was anonymous, no informed consent was required. Our study was conducted based on the guidelines set by the Declaration of Helsinki [9] and the Health Insurance Portability and Accountability (HIPAA) [10]. Furthermore, our results are reported according to the STROBE statement [11].

### 2.2. Study Population

We screened all consecutive patients who were admitted to the Department of Respiratory Medicine of Larissa University Hospital during our study period. We included patients 18 years old or older, with severe COVID-19 infection. COVID-19 infection was diagnosed based on a positive real-time polymerase chain reaction (PCR), which was performed on a nasopharyngeal swab. A severe COVID-19 infection was defined as any of the following: (1) respiratory rate of 30 or more breaths per minute, (2) oxygen saturation (SpO_2_) equal or less than 93% on ambient air or a ratio of arterial oxygen partial pressure to fractional inspired oxygen (PaO_2_/FiO_2_) equal or less than 300 mmHg [12]. Patients were treated according to the COVID-19 treatment guidelines of the National Institute of Health (NIH) [12]. We excluded patients with autoimmune disorders and patients with malignancy and recent chemotherapy.

### 2.3. Data Collection and Definitions

We collected the following data for all included patients: demographic data, past medical history, medications, vital signs on admission (oxygen saturation, blood pressure, heart rate, PaO_2_/FiO_2_ ratio) and length and outcome of hospitalization (LOH). Outcomes were defined as use of high-flow nasal cannula (HFNC), admission to Intensive Care Unit (ICU) or death during hospitalization. Moreover, the following laboratory values were recorded on admission; white blood cells count (WBC), neutrophil count (N), lymphocyte count (L), platelet count (PLTs) and C-reactive protein (CRP).

Additionally, the following ratios based on-admission values were calculated: CRP to lymphocyte ratio (CLR), lymphocyte to neutrophil ratio (LNR) and derived variation of neutrophil to lymphocyte ratio (dv-NLR (neutrophil/white blood count-lymphocyte)).

Following this, based on multivariate analysis, the optimal cut-off points of on admission values of C-reactive protein (CRP), CRP to lymphocyte ratio (CLR), lymphocyte to neutrophil ratio (LNR) and derived variation of neutrophil to lymphocyte ratio (dv-NLR (neutrophil/white blood count-lymphocyte)) for each predetermined outcome (use of HFNC, admission to ICU, death) were indicated. Based on which biomarker was found to be a predictor for each outcome and on the cut-off points, the following combination scores were defined:

- HFNC: Based on the cut-off of CRP, LNR, dv-NLR and CLR, which were found to be predictors for HFNC, 3 scores were defined: CRP and LNR (C-CRP #1), CRP and dv-NLR (C-CRP #2), CRP and CLR (C-CRP #3) (Table 1).
- Death: Based on the cut-off of CRP and CLR, which were found to be predictors for death, the score of CRP and CLR (C-CRP #3*) was defined (Table 2).

Of note, none of the studied biomarkers was predictive for the ICU admission in our study sample, so no further analysis was performed.

The final combination scores were classified as normal or elevated as follows (Table 3 and Table 4): 2 points (if both scores were elevated); 1 point (if one of two scores was elevated) and 0 points (if no score was elevated).

### 2.4. Statistical Analysis

We used means or median values with standard deviation (SD) or interquartile range (IQR) to present continuous data with normal and non-normal distribution based on the Shapiro–Wilk test, respectively. Nominal and ordinal data are presented as counts and percentages. Univariate analysis was conducted for all study endpoints. Our results are presented as Odds Ratio (OR) and 95% confidence interval (95%CI). For dichotomous and continuous data, logistic and linear regressions were conducted, respectively. Diagnostic accuracy was estimated using receiver-operating characteristic (ROC) curves. Sensitivity (sens) and Specificity (spe) as well as the Area Under the Curve (AUC) was calculated for all curves. The optimal cut-off value was estimated as the value offering the highest possible sens and spe. Binomial logistic regression analysis was used to establish the predictive role for each biomarker. The level of statistical significance was set at *p* < 0.05. An author trained in biostatistics carried out all statistical analyses using the statistical environment R [13].

## 3. Results

### 3.1. Basic Characteristics of Our Study Sample

Overall, 115 consecutive patients (69 males, 60%), with a mean age of 57.7 ± 16.3 years and suffering from severe COVID-19 were admitted to our hospital during the study period. The mean length of hospital stay was eight days (standard deviation ±4.5). Arterial hypertension (47 patients, 40.9%) and hyperlipidaemia (36 patients, 31.1%) constituted the most common registered comorbidities. The mean systolic arterial pressure and heart rate on admission were 124.8 (IQR: 12.8) mmHg and 79 (IQR: 13) beats/min, respectively. Based on the PaO_2_/FiO_2_ values, almost half of the patients (*n* = 58) presented with PaO_2_/FiO_2_ values (<300, IQR: 50.4). Of note, none of our patients was vaccinated against COVID-19. Ultimately, 37 (32.2%) patients required escalation of respiratory support to HFNC, eight patients (7%) were admitted to the ICU, and nine patients (7.8%) died.

### 3.2. Combined Scores and HFNC

**Figure 1:**
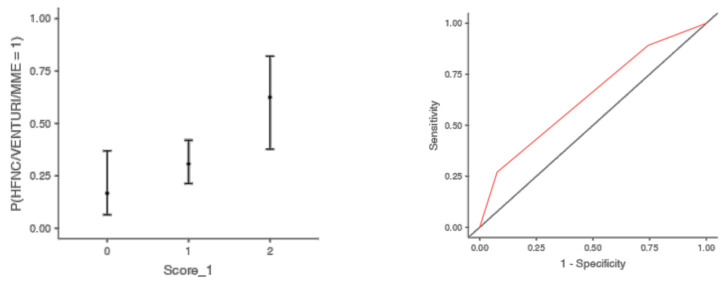
Two points of C-CRP #1 predicted the use of HFNC in patients suffering from severe COVID-19 infection.

**Figure 2:**
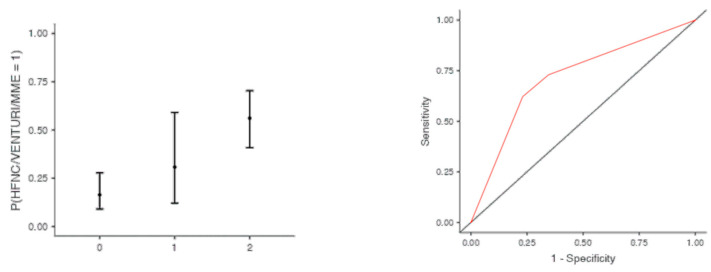
Two points of C-CRP #3 predicted the use of HFNC in patients suffering from severe COVID-19 infection.

**Figure 3:**
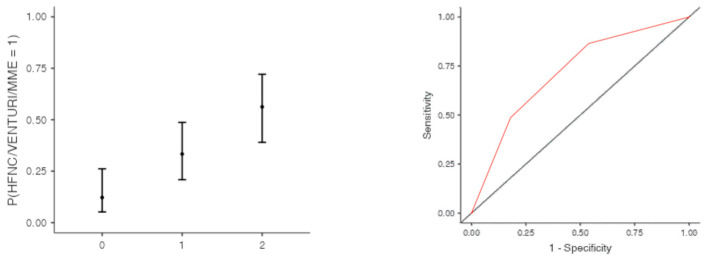
One and two points of C-CRP #2 predicted the use of HFNC in patients suffering from severe COVID-19 infection.

The optimal cut off point was 3.2 for CRP (AUC: 0.740; sens: 0.702; spec 0.705), 0.231 for LNR (AUC: 0.367; sens: 0.46; spec 0.46), 0.90 for dv-NLR (AUC: 0.678; sens: 0.594; spec 0.60) and 0.004 for CLR (AUC: 0.742; sens: 0.65; spec 0.731) (Table 5). As far as the C-CRP #1 score is concerned, two points of C-CRP #1 with an AUC of 0.640 (sens: 0.923; spec 0.270) (Figure 1)) predicted the use of HFNC with a probability as high as 0.625 (*p* = 0.005). However, 1 point of C-CRP #1 did not exhibit any prognostic value at all for escalation of respiratory support with HFNC. As for C-CRP #3, 2 points of C-CRP #3 with an AUC of 0.715 (sens: 0.622, spec 0.769) (Figure 2) predicted the use of HFNC with a probability as high as 0.561 (*p* < 0.001). One point of C-CRP #3 did not exhibit any prognostic value for HFNC use. On the other hand, both 1 and 2 points of C-CRP #2 predicted the need for HFNC. One point of C-CRP #2 with an AUC of 0.717 (sens: 0.821; spec 0.486; *p* = 0.027) and 2 points of C-CRP #2 (*p* < 0.001) predicted the need for HFNC with a probability of 0.333 and 0.562, respectively (Figure 3).

### 3.3. Combined Scores and Death

**Figure 4:**
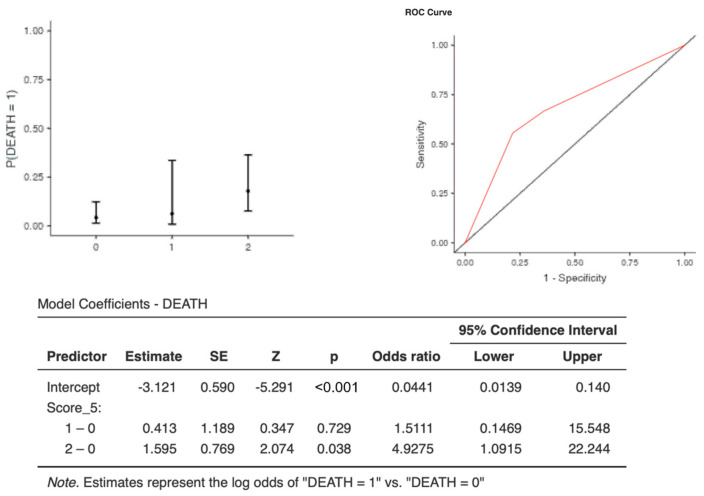
Two points of C-CRP #3* predicted death in patients suffering from severe COVID-19 infection.

The optimal cut-off points for each biomarker were as follows: CRP (AUC: 0.673, sens: 0.67, spec 0.75, cut-off 1.11) and CLR (AUC: 0.720, sens: 0.66, spec 0.77, cut-off 3.2*1033) (Table 6). Interestingly, although one point of C-CRP #3* was not predictive for death, two points of C-CRP #3* (OR, 4.92; 95%CI, 1.09–22.24) with an AUC: 0.681, (sens: 0.00, spec 1.00) and an accuracy of 0.922 predicted death (*p* = 0.0038) in COVID-19 patients (Table 6 and Table 7 & Figure 4).

## 4. Discussion

Based on the results of our study it seems that the combination scores of simple inflammatory biomarkers based on admission values of patients with COVID-19 infection could serve as valuable assets in predicting the need for escalation of respiratory support with the use of HFNC and mortality in severe infection, respectively. Most previous studies have independently investigated the role of several biomarkers and have assessed their clinical significance in patients suffering from severe COVID-19 infection [2,14]. To the best of our knowledge, this is the first study to investigate and determine the prognostic value, in terms of escalation of respiratory support and mortality, of admission values and combination scores of simple inflammatory biomarkers in patients with severe COVID-19 infection.

Although the vast majority of patients suffering from COVID-19 infection have a good prognosis, some patients may suffer from severe disease with the need for hospitalization, escalation of respiratory support and/or ICU admission. A percentage of the aforementioned patients may even experience multiple organ dysfunction syndrome (MODS) and death [15]. However, most of patients who will eventually experience MODS or death will initially present with mild manifestations, such as mild fever, cough or muscle soreness, in the early stages of the disease [15]. Their condition will deteriorate acutely either in the later stages of the disease or during recovery [15]. ARDS, MODS and even death can occur rapidly as a consequence/manifestation of the excessive and prolonged cytokine storm, which holds a fundamental role in disease aggravation [15]. Hence, experts suggest that timely and early control of the cytokine storm may be the key in delaying physiological deterioration, improving treatment success rate and reducing the mortality rate of patients with severe COVID-19 [15].

Based on the idea described by Hirahara et al. [16] and by Zhu et al. [17] regarding the prognosis of patients with advanced cancer and exacerbation of asthma, respectively, we investigated the utility of combination biomarkers, based on admission values, in predicting the prognosis of patients suffering from severe COVID-19 disease. Although the current literature [3,18,19,20,21,22] suggests an association between several, mostly hemogram-derived ratios and COVID-19 disease prognosis, experts [21,22] suggest that further research is needed to identify the best-fit predictive biomarker and that the heterogeneity of patients with COVID-19 highlights the need for implementation of multiple biomarkers in order to ideally evaluate the dynamic course of the disease. Over the progression of the pandemic, several studies [5,23,24,25] have pointed out the utility of CRP in differentiating COVID-19 patients with or without pneumonia and/or with a higher incidence of complications and worse overall prognosis [5,8,23,24,25]. Moreover, Çakirca et al. [5], in a large study with 306 patients, showed that the CLR index should be considered the best predictor of pneumonia in COVID-19 patients. Thus, we used combination scores of admission values to find the best fitting combination biomarker in order to predict the need for escalation of respiratory support with HFNC and death in patients with severe COVID-19 infection. We followed the scoring system of zero, one and two points as proposed by Hirahara et al. [16] and by Zhu et al. [17].

Interestingly, two points of the combination score of CRP and CLR was found to be a predictor for two of the outcomes of our study, escalation of respiratory support with the use of HFNC and death. As far as death is concerned, the accuracy of the predictive value of the combination score of CRP and CLR was quite high (0.922) with a specificity of 1 and sensitivity of zero. This could have significant utility in everyday clinical practice as it indicates that every patient with a combination score of CRP and CLR of two points (cut-off values: CRP > 1.1 and CLR > 3.2*1033) is at high risk of death. On the other hand, scoring two points using the combination score of CRP and CLR with the following cut-off values (CRP > 3.2 and CLR > 0.004), higher CRP but lower CLR, when compared to death, could help in identifying patients that would eventually need HFNC therapy.

Nevertheless, regarding escalation of respiratory support with HFNC both one and two points of C-CRP #2 (combination score of CRP and dv-NLR) predicted the use of HFNC in patients suffering from severe COVID-19 infection. With an accuracy of 0.713 and a probability of 0.33 and 0.562 for 1 and 2 points respectively, score C-CRP #2 could potentially serve as a useful prognostic factor for the selection of patients in need of closer monitoring and advanced treatment in terms of respiratory support.

The need for optimal and early risk stratification of COVID-19 patients seems to be of utmost prominence. The described biomarkers of the present study only require some milliliters of whole blood and they are cheap, easy and simple to calculate. They could serve as valuable assets in early triage of high-risk patients, help in choosing the optimal treatment regimen and preventing severe COVID-19 infection complications. However, the present study has some limitations. It is a retrospective study with a relatively small number of patients from a single center during a certain period of the pandemic. Nevertheless, all the data were prospectively collected, hence we did not have any missing patients or missing values. Second, we did not use any of the described advanced biomarkers, such as interleukins, in an attempt to only investigate widely available and routinely performed biomarkers that are simple to calculate, inexpensive and fast to obtain. Last, although all blood samples were obtained upon admission, there is no guarantee that all patients arrived on the same day from symptom onset. Larger, multicenter studies seem to be worthwhile in order to optimally stratify severe COVID-19 infection upon admission and pinpoint the patients that require aggressive treatment and close monitoring.

## 5. Conclusions

The combination scores of CRP and inflammatory biomarkers, based on admission values, are promising predictors of respiratory support escalation using HFNC and of mortality, in patients suffering from severe COVID-19 infection. In our study two points of C-CRP #1 and two points of C-CRP #3 predicted HFNC with a high probability. Moreover, both one and two points of C-CRP #2 predicted the escalation of respiratory support with HFNC. As far as death is concerned, two points of C-CRP #3* predicted death with a very high accuracy in severe COVID-19 infection. However, a comprehensive evaluation in large, multicenter studies seems mandatory in order to establish the clinical utility of these biomarkers and to draw generalizable conclusions.

## Figures and Tables

**Table 1:** Definitions of combined scores for HFNC.

C-CRP #1	CRP + LNR
C-CRP #2	CRP + dv-NLR
C-CRP #3	CRP + CLR

**Table 2:** Definitions of combined scores for death.

CLR	CRP/Lymphocytes
C-CRP #3*	CRP + CLR

**Table 3:** Final combination scores for HFNC.

0 point	CRP < AUC and LNR < AUC, or CRP < AUC and dv-NLR < AUC, or CRP < AUC and CLR < AUC
1 point	CRP < AUC and LNR > AUC, or CRP > AUC and LNR < AUC, or CRP > AUC and dv-NLR < AUC, or CRP < AUC and dv-NLR > AUC, or CRP < AUC and CLR > AUC, or CRP > AUC and CLR < AUC
2 points	CRP > AUC and LNR > AUC, or CRP > AUC and dv-NLR > AUC, or CRP > AUC and CLR > AUC

**Table 4:** Final combination scores for death.

0 point	CRP < AUC + CLR < AUC
1 point	CRP < AUC + CLR > AUC, or CLR < AUC + CRP > AUC
2 points	CRP > AUC + CLR > AUC

**Table 5:** Optimal cutoff points of biomarkers for HFNC.

Value	Cutoff Point	AUC	Sens/Spec
CRP	3.2	0.740	0.702/0.705
LNR	0.231	0.367	0.46/0.46
dv-NLR	0.90	0.678	0.594/0.60
CLR	0.004	0.751	0.622/0.769

**Table 6:** Optimal cutoff points of biomarkers for death.

Value	Cutoff Point	AUC	Sens/Spec
CRP	1.11	0.673	0.673/0.75
CLR	3.2*1033	0.720	0.66/0.77

**Table 7:** Prediction of C-CRP #3* for death.

Predictive Measures			
Accuracy	Specificity	Sensitivity	AUC
0.922	1.00	0.00	0.681

## Data Availability

The data presented in this study are available on request from the corresponding author. The data are not publicly available due to ethics and general data protection regulation.

## References

[1] Qin C., Zhou L., Hu Z., Zhang S., Yang S., Tao Y., Xie C., Ma K., Shang K., Wang W. (2020). Dysregulation of Immune Response in Patients with Coronavirus 2019 (COVID-19) in Wuhan, China. Clin. Infect. Dis..

[2] Yılmaz A., Taşkın Ö., Demir U., Soylu V.G. (2023). Predictive Role of Biomarkers in COVID-19 Mortality. Cureus.

[3] Citu C., Gorun F., Motoc A., Sas I., Gorun O.M., Burlea B., Tuta-Sas I., Tomescu L., Neamtu R., Malita D. (2022). The Predictive Role of NLR, d-NLR, MLR, and SIRI in COVID-19 Mortality. Diagnostics.

[4] Aly M.M., Meshref T.S., Abdelhameid M.A., Ahmed S.A., Shaltout A.S., Abdel-Moniem A.E., Hamad D.A. (2021). Can Hematological Ratios Predict Outcome of COVID-19 Patients? A Multicentric Study. J. Blood Med..

[5] Damar Çakırca T., Torun A., Çakırca G., Portakal R.D. (2021). Role of NLR, PLR, ELR and CLR in differentiating COVID-19 patients with and without pneumonia. Int. J. Clin. Pract..

[6] Yang A.P., Liu J.P., Tao W.Q., Li H.M. (2020). The diagnostic and predictive role of NLR, d-NLR and PLR in COVID-19 patients. Int. Immunopharmacol..

[7] Zhang J.N., Gao Y., Wang X.T., Li N.N., Du X., Tang Y.J., Lai Q.Q., Chen P.F., Yue C.S., Wu J.H. (2022). Lymphocyte-C-reactive protein ratio can differentiate disease severity of COVID-19 patients and serve as an assistant screening tool for hospital and ICU admission. Front. Immunol..

[8] Ntalouka M.P., Pantazopoulos I., Brotis A.G., Pagonis A., Vatsiou I., Chatzis A., Rarras C.N., Kotsi P., Gourgoulianis K.I., Arnaoutoglou E.M. (2022). Prognostic role of simple inflammatory biomarkers in patients with severe COVID-19: An observational study. Hippokratia.

[9] World Medical Association (2013). World Medical Association Declaration of Helsinki: Ethical principles for medical research involving human subjects. JAMA.

[10] Office of the Assistant Secretary for Planning and Evaluation Health Insurance Portability and Accountability Act of 1996. https://aspe.hhs.gov/reports/health-insurance-portability-accountability-act-1996.

[11] Von Elm E., Altman D.G., Egger M., Pocock S.J., Gøtzsche P.C., Vandenbroucke J.P., STROBE Initiative (2014). The Strengthening the Reporting of Observational Studies in Epidemiology (STROBE) Statement: Guidelines for reporting observational studies. Int. J. Surg..

[12] National Institute of Health Coronavirus Disease 2019 (COVID-19). Treatment Guidelines. https://www.covid19treatmentguidelines.nih.gov/.

[13] The R Foundation The R Project for Statistical Computing. https://www.r-project.org/.

[14] Ponti G., Maccaferri M., Ruini C., Tomasi A., Ozben T. (2020). Biomarkers associated with COVID-19 disease progression. Crit. Rev. Clin. Lab. Sci..

[15] Ye Q., Wang B., Mao J. (2020). The pathogenesis and treatment of the ‘Cytokine Storm’ in COVID-19. J. Infect..

[16] Hirahara T., Arigami T., Yanagita S., Matsushita D., Uchikado Y., Kita Y., Mori S., Sasaki K., Omoto I., Kurahara H. (2019). Combined neutrophil-lymphocyte ratio and platelet-lymphocyte ratio predicts chemotherapy response and prognosis in patients with advanced gastric cancer. BMC Cancer.

[17] Zhu X., Zhou L., Li Q., Pan R., Zhang J., Cui Y. (2021). Combined score of C-reactive protein level and neutrophil-to-lymphocyte ratio: A novel marker in distinguishing children with exacerbated asthma. Int. J. Immunopathol. Pharmacol..

[18] Zhu B., Feng X., Jiang C., Mi S., Yang L., Zhao Z., Zhang Y., Zhang L. (2021). Correlation between white blood cell count at admission and mortality in COVID-19 patients: A retrospective study. BMC Infect. Dis..

[19] Liu K., Fang Y.Y., Deng Y., Liu W., Wang M.F., Ma J.P., Xiao W., Wang Y.N., Zhong M.H., Li C.H. (2020). Clinical characteristics of novel coronavirus cases in tertiary hospitals in Hubei Province. Chin. Med. J..

[20] Zhang M.Q., Wang X.H., Chen Y.L., Zhao K.L., Cai Y.Q., An C.L., Lin M.G., Mu X.D. (2020). Clinical features of 2019 novel coronavirus pneumonia in the early stage from a fever clinic in Beijing. ZhonghuaJie He He Hu Xi Za Zhi.

[21] Ghobadi H., Mohammadshahi J., Javaheri N., Fouladi N., Mirzazadeh Y., Aslani M.R. (2022). Role of leukocytes and systemic inflammation indexes (NLR, PLR, MLP, dNLR, NLPR, AISI, SIR-I, and SII) on admission predicts in-hospital mortality in non-elderly and elderly COVID-19 patients. Front. Med..

[22] Gutiérrez-Pérez I.A., Buendía-Roldán I., Pérez-Rubio G., Chávez-Galán L., Hernández-Zenteno R.J., Aguilar-Duran H., Fricke-Galindo I., Zaragoza-García O., Falfán-Valencia R., Guzmán-Guzmán I.P. (2022). Outcome predictors in COVID-19: An analysis of emergent systemic inflammation indices in Mexican population. Front. Med..

[23] Malik P., Patel U., Mehta D., Patel N., Kelkar R., Akrmah M., Gabrilove J.L., Sacks H. (2021). Biomarkers and outcomes of COVID-19 hospitalisations: Systematic review and meta-analysis. BMJ Evid. Based Med..

[24] Tjendra Y., Al Mana A.F., Espejo A.P., Akgun Y., Millan N.C., Gomez-Fernandez C., Cray C. (2020). Predicting Disease Severity and Outcome in COVID-19 Patients: A Review of Multiple Biomarkers. Arch. Pathol. Lab. Med..

[25] Bivona G., Agnello L., Ciaccio M. (2021). Biomarkers for Prognosis and Treatment Response in COVID-19 Patients. Ann. Lab. Med..

